# Rhodomyrtone: a new anti-*Staphylococcus aureus* agent against resistant strains

**DOI:** 10.1093/jacamr/dlae097

**Published:** 2024-07-03

**Authors:** Louis D Saravolatz, Joan Pawlak

**Affiliations:** Department of Medicine, Ascension-St John Hospital, Grosse Pointe Woods, MI, USA; Department of Medicine, Thomas Mackey Center for Infectious Disease Research, Grosse Pointe Woods MI, USA; Department of Medicine, Wayne State University School of Medicine, Detroit, MI, USA; Department of Medicine, Ascension-St John Hospital, Grosse Pointe Woods, MI, USA; Department of Medicine, Thomas Mackey Center for Infectious Disease Research, Grosse Pointe Woods MI, USA; Department of Medicine, Wayne State University School of Medicine, Detroit, MI, USA

## Abstract

**Background:**

Rhodomyrtone is a novel plant-derived antibiotic compound originally isolated from *Rhodomyrtus tomentosa* leaf extract.

**Objectives:**

To evaluate the activity of rhodomyrtone against a group of MRSA strains, including isolates with reduced susceptibility or resistance to vancomycin, daptomycin, linezolid and ceftaroline.

**Methods:**

Broth microdilution testing was used to determine the MICs and MBCs of rhodomyrtone, fosfomycin, vancomycin, daptomycin, linezolid and ceftaroline.

**Results:**

ROM had an MIC_90_ of 1 mg/L against 110 strains of MRSA from blood isolates as well as for all other isolates that were daptomycin resistant, VISA, VRSA or LRSA. The MBC_90_ were 4 mg/L across all groups tested. Among all *S. aureus* groups tested the ROM MBC did not exceed 8 mg/L.

**Conclusions:**

Rhodomyrtone demonstrated excellent activity against MRSA as well as isolates with resistance or reduced activity to other anti-MRSA drugs including vancomycin, daptomycin and linezolid. Rhodomyrtone may have potential clinical utility when treating patients with infections caused by MRSA including those with reduced susceptibility to first-line anti-MRSA antimicrobial agents.

## Introduction

Rhodomyrtone (ROM) is a novel plant-derived antibiotic compound originally isolated from *Rhodomyrtus tomentosa* leaf extract with activity against methicillin resistant *Staphylococcus aureus* (MRSA). ROM can inhibit multiple sites including macromolecular biosynthesis such as DNA, RNA, proteins, the cell wall and lipid.^1^ In addition, ROM has been shown to interfere with the expression of virulence genes of *Staphylococcus aureu*s including the ability to inhibit direct interaction with phospholipid suggesting that one of the targets is the cytoplasmic membrane.^2^ ROM targets the cell membrane by causing the dissipation of the membrane potential and release of ATP and cytoplasmic proteins. Resistance may occur based on a point mutation in the farR regulatory chain causing an overexpression of FarE that probably acts as a phospholipid efflux pump responsible for the resistance to ROM.^3^ These multiple targets against MRSA make this a representative agent of a class of antibacterial agents to be considered in the management of methicillin or other drug-resistant Gram-positive bacteria.

The Infectious Diseases Society of America guidelines for treatment of *S. aureus* infections have suggested there is limited efficacy along with concern for the emergence of resistance of current antibiotic therapy with either vancomycin or daptomycin.^4^ Thus, the need for new classes of antimicrobial agents may provide treatment options for *S. aureus* infections resistant to current available agents.

The purpose of this study was to evaluate the activity of ROM as a representative of a new class of antibiotics, compared to other agents against a group of MRSA strains, including isolates with reduced susceptibility or resistance to currently available anti-MRSA antibiotics including vancomycin, daptomycin, linezolid, fosfomycin and ceftaroline.

## Material and methods

A collection of *S. aureus* isolates was selected for evaluation. Vancomycin intermediate *S. aureus* (VISA) isolates (*n* = 31), vancomycin-resistant *S. aureus* (VRSA) isolates (*n* = 15) and linezolid-resistant *S. aureus* (LRSA) isolates (*n* = 4) were obtained from the Network on Antimicrobial Resistance in *Staphylococcus aureus* (NARSA). NARSA is now supported by and known as BEI Resources. Two additional LRSA isolates were obtained from Robinson Memorial Hospital in Ravenna, OH, USA. Also included in the study were 110 blood isolates, four VISA isolates and 14 daptomycin-resistant *S. aureus* (DRSA) isolates obtained from patients admitted to Ascension St John Hospital Detroit, MI, USA. Some isolates met criteria for both VISA and DRSA and thus were included in both categories. These organisms have been characterized in terms of their molecular characteristics that are included in the Supplementary Tables S1–S11(available as Supplementary data at *JAC-AMR* Online).

Broth microdilution testing using CAMHB was used to determine the MICs of ROM, fosfomycin, vancomycin, daptomycin, linezolid and ceftaroline. For all testing with fosfomycin, the CAMHB was supplemented with α-D-glucose-6-phosphate to a final concentration of 25 mg/L.^5^ When testing daptomycin, CAMHB was supplemented with 50 mg/L calcium.^6^ All antibiotic powders were purchased from Sigma-Aldrich (St Louis, MO, USA). ROM powder (≥95% purity), was derived from the *Rhodomyrtus tomentosa* plant. The ROM powder was suspended in 100% dimethyl sulfoxide (DMSO) and stored at −80°C until use. MICs were determined in accordance with the CLSI guidelines. The plates were prepared in house and inoculated with approximately 5 × 105 cfu/mL of each organism.^7^ The plates were incubated at 35°C in ambient air for 16–24 h. MICs were read visually as the lowest drug concentration well with no visible bacterial growth. *S. aureus* ATCC 29213 was used daily to monitor quality control for all the agents tested. This ATCC 29213 is an established methicillin susceptible S. aureus used to demonstrate reproducibility of our results and to show activity against *S. aureus* that is methicillin susceptible. After primary incubation, MICs were recorded, and MBCs were determined by spotting 10 mL from wells at and above MICs on drug-free agar medium. The cfu results from sampled test wells were used to determine MBCs that caused ≥3 log bacterial killing. MBCs were determined according to CLSI guidelines.^8^

All testing was performed at three separate times, with all MIC results were within two doubling dilutions when repeated during the three different experiments. The result reported required agreement on two of the three runs performed as shown in the Supplementary Tables S1–S11. Although this was the criteria used, the results were always within one dilution of each other. CLSI does not publish breakpoints for ROM or fosfomycin. We used the EUCAST breakpoints to determine the breakpoints of daptomycin, linezolid and ceftaroline. EUCAST does not currently have breakpoints for fosfomycin against *Staphylococcus aureus* as antimicrobial susceptibility testing is discouraged.^9^ There is no evidence to suggest that MICs of fosfomycin against *Staphylococcus aureus* are predictive of clinical efficacy. We used CLSI breakpoints for vancomycin to maintain our original VISA classifications consistent with our previous work with these agents.^5,6^

In Table 1, the percentage susceptible and resistant classifications for vancomycin, daptomycin, linezolid and ceftaroline are consistent with EUCAST breakpoints.

**Table 1.** *In vitro* activity of rhodomyrtone, fosfomycin, vancomycin, daptomycin, linezolid and ceftaroline against study isolates

	MIC (mg/L)					MBC (mg/L)		
Isolate and agent	Range	MIC_50_	MIC_90_	%S^a^	%R	Range	MBC_50_	MBC_90_
MRSA blood (*n* = 110)								
Rhodomyrtone	1	1	1	NA	NA	1–8.0	2	4
Fosfomycin^c^	1.0–16	4	8	NA	NA	ND	ND	ND
Vancomycin	0.5–4.0	1	1	99	1	0.5–4.0	1	2
Daptomycin	0.25–4.0	0.5	1	96	4	0.25–4.0	0.5	1
Linezolid	1.0–4.0	2	2	100	0	8.0 to >8.0	>8	>8
Ceftaroline	0.25–2.0	0.5	1	99	1	0.25–2.0	0.5	1
DRSA (*n* = 40)								
Rhodomyrtone	0.5–1.0	1	1	NA	NA	1.0–8.0	2	4
Fosfomycin	1.0 to >512	4	32	NA	NA	1.0 to >512	8	64
Vancomycin	1.0–8.0	4	8	35	65	1.0–8.0	4	8
Daptomycin	2.0–8.0	2	4	0	100	2.0–16	2	4
Linezolid	1.0–2.0	2	2	100	0	4.0 to >8	>8	>8
Ceftaroline	0.25–1.0	0.5	1	100	0	0.25–2.0	1	1
VISA (*n* = 35)								
Rhodomyrtone	0.5–1.0	1	1	NA	NA	1.0–8.0	2	4
Fosfomycin	1.0 to >512	8	>512	NA	NA	1.0 to >512	16	>512
Vancomycin	4.0–8.0	4	8	0	100	4.0–8.0	4	8
Daptomycin	0.5–8.0	2	4	26	74	1.0–16	2	4
Linezolid	1.0–4.0	2	2	100	0	4.0 to >8.0	8	>8
Ceftaroline	0.25–1.0	0.5	1	100	0	0.25–2.0	1	2
VRSA (*n* = 15)^b^								
Rhodomyrtone	0.5–1.0	1	1	NA	NA	1.0–4.0	2	4
Fosfomycin	2.0–16	4	8	NA	NA	2.0–32	8	32
Vancomycin	32 to >64	>64	>64	0	100	64 to >64	>64	>64
Daptomycin	0.25–1.0	0.5	1	100	0	0.25–1.0	0.5	1
Linezolid	1.0–4.0	2	2	100	0	4.0 to >8.0	8	>8
Ceftaroline	0.25–1.0	0.5	1	100	0	0.25–1.0	1	1
LRSA (*n* = 6)								
Rhodomyrtone	0.5–1.0	1	NC	NA	NA	1.0–4.0	2	NC
Fosfomycin	8.0–32	5	NC	NA	NA	8.0–32	16	NC
Vancomycin	1.0–2.0	1	NC	100	0	1.0–2.0	1	NC
Daptomycin	0.5–1.0	0.5	NC	100	0	0.5–1.0	0.5	NC
Linezolid	>8	>8	NC	0	100	>8	>8	NC
Ceftaroline	0.5–1.0	1	NC	100	0	1	1	NC

S, susceptible; R, resistant; ND, not done; NC, not calculated because there are fewer than 10 isolates in category; NA, breakpoints not available.

^a^Guideline used to interpret the susceptibility categories were based on EUCAST v.14.0, 2024 publication reference.^9^

^b^Guideline used to classify the isolates into VISA were based on CLSI performance standards reference.^6^

^c^Fosfomycin was tested by broth microdilution reference.^5^

## Results

The activity of ROM along with comparator agents against tested isolates is provided in Table 1. ROM demonstrated an MIC_90_ of 1 mg/L against 110 strains of MRSA from blood isolates. The MIC_90_ was also 1 mg/L for all other isolates that were daptomycin resistant, VISA, VRSA or LRSA. The MBC_90_ were 4 mg/L across all groups tested but did range from 1 to 8 mg/L. Since the MBCs were usually two to four times higher than the MIC, these data indicate that ROM is bactericidal against *S. aureus*. There are no EUCAST or CLSI breakpoints for ROM at this time and thus the ROM percentage susceptible and resistant cannot be reported in this study. The testing of ROM against the ATCC 29213 know methicillin susceptible *S. aureus* was consistently 1 mg/L on all the performed 15 runs (see Supplementary Data).

## Discussion

The *in vitro* activity described in this study demonstrates that ROM has good *in vitro* activity against strains of MRSA resistant to daptomycin, VISA, VRSA and linezolid. Previous work by Leejae *et al.* suggested that ROM had activity against MRSA, but these investigators only studied five MRSA strains and two VISA strains.^10^ The results from Leejae and colleagues showed an MIC of 0.5–1.0 mg/L for the seven strains tested and no difference between the MRSA and VISA strains. These results are consistent with our observations. In addition, time kill curves against MRSA have been published and indicated that the microorganism was assessed for survivability after exposure to ROM at eight times the MIC for 10 h, and four and two times the MIC for 24 h.^10^ This suggests bactericidal activity from these experiments. Also, when ROM was studied against enterococci including vancomycin-resistant enterococci and an *E. faecalis* ATCC 29212 strain, ROM demonstrated bacteriostatic activity against the enterococcus.^10^

There is a potential for ROM resistance acquisition, given that a single FabR mutation is sufficient to induce high-level resistance. However, this did not show any change in susceptibility to 21 other antimicrobial agents tested when comparing the native strain to the RomR mutant strain.^3^ The likelihood of this mutant emerging and frequency is unknown and the impact of combination therapy on delaying this emergence of resistance will need further evaluation.

At this time, there is insufficient clinical data to suggest achievable serum levels and what appropriate breakpoints would be to determine whether resistance can occur. However, ROM has been used in the clinical trial in the management of acne vulgaris.^11^ This clinical trial applied topical ROM and compared 1% liposomal encapsulated ROM serum compared to a marketed 1% clindamycin gel. In a randomized double-blind controlled clinical trial of 60 volunteers a significant reduction in the total number of acne lesions was demonstrated in both treatment groups at weeks 2 and 8. In addition, at the end of the trial the total number of inflamed acne counts in the ROM serum group were significantly reduced compared to the clindamycin treated group. Noteworthy is that subjects demonstrated no signs of irritation or side effects from the application of the 1% liposomal encapsulated ROM serum.

There is limited clinical experience with the application of ROM by any route currently. Although it does appear that ROM may represent a novel class of drugs, with multiple targeted sites for activity, while demonstrating *in vitro* activity against strains resistant to multiple classes of drugs including those commonly used as anti-MRSA drugs such as daptomycin, vancomycin and linezolid.

### Conclusion

ROM consistently demonstrated good inhibitory and bactericidal activity against MRSA strains resistant to commonly available anti-MRSA drugs in this study. The activity of ROM provides the potential for an entire new class of drugs to be utilized against MRSA in the future.

## Supplementary Material

dlae097_Supplementary_Data

## Supplementary data

Tables S1–S11 are available as Supplementary Data at *JAC-AMR* Online.
