# Supplementary material for: Rhodomyrtone: a new anti-Staphylococcus aureus agent against resistant strains
Source: JAC Antimicrob Resist. 2024 Jul 3;6(4):dlae097. doi: 10.1093/jacamr/dlae097 (PMC11220652; doi:10.1093/jacamr/dlae097)
Supplement: dlae097_Supplementary_Data [file dlae097_supplementary_data.docx]

Supplemental Table 1: Molecular Characteristics, Source and In Vitro Activity of Rhodomyrtone, Fosfomycin, Vancomycin, Daptomycin, Ceftaroline and Linezolid against each MRSA study isolate (n=110).

| ID # | ROM | | FOS | VAN | | DAP | | CPT | | LZD | | SCC | PVL | arc(A) | Source | PFGE Group |
| --- | --- | --- | --- | --- | --- | --- | --- | --- | --- | --- | --- | --- | --- | --- | --- | --- |
|  | MIC | MBC | MIC | MIC | MBC | MIC | MBC | MIC | MBC | MIC | MBC |  |  |  |  |  |
| MRSA-79 | 1 | 8 | 4 | 1 | 1 | 0.5 | 0.5 | 1 | 1 | 2 | >8 | II | Neg | Neg | Blood | USA-100 |
| MRSA-80 | 1 | 2 | 4 | 0.5 | 0.5 | 0.25 | 0.25 | 0.5 | 0.5 | 2 | >8 | IV a | Pos | Pos | Blood | USA-300 |
| MRSA-81 | 1 | 2 | 2 | 1 | 1 | 0.5 | 0.5 | 1 | 1 | 2 | >8 | II | Neg | Neg | Blood | USA-100 |
| MRSA-82 | 1 | 2 | 2 | 2 | 2 | 0.5 | 1 | 0.5 | 0.5 | 2 | >8 | IV a | Pos | Pos | Blood | USA-300 |
| MRSA-83 | 1 | 2 | 8 | 1 | 1 | 0.5 | 0.5 | 1 | 1 | 2 | >8 | II | Neg | Neg | Blood | USA-100 |
| MRSA-84 | 1 | 2 | 8 | 1 | 1 | 0.5 | 0.5 | 0.5 | 0.5 | 2 | >8 | IV a | Pos | Pos | Blood | USA-300 |
| MRSA-85 | 1 | 4 | 8 | 1 | 1 | 0.5 | 0.5 | 1 | 1 | 2 | >8 | II | Neg | Neg | Blood | USA-100 |
| MRSA-86 | 1 | 2 | 8 | 1 | 1 | 0.5 | 0.5 | 0.5 | 1 | 2 | >8 | IV a | Pos | Pos | Blood | USA-300 |
| MRSA-87 | 1 | 2 | 2 | 1 | 1 | 0.5 | 0.5 | 0.25 | 0.25 | 2 | >8 | IV a | Pos | Pos | Blood | USA-300 |
| MRSA-88 | 1 | 2 | 2 | 1 | 1 | 0.5 | 0.5 | 0.5 | 1 | 2 | >8 | IV | Neg | Neg | Blood | No Match |
| MRSA-89 | 1 | 2 | 4 | 1 | 2 | 0.5 | 0.5 | 0.5 | 1 | 2 | >8 | IV a | Pos | Pos | Blood | USA-300 |
| MRSA-90 | 1 | 2 | 2 | 1 | 1 | 0.5 | 0.5 | 0.5 | 0.5 | 1 | >8 | IV a | Neg | Pos | Blood | USA-300 |
| MRSA-91 | 1 | 4 | 4 | 1 | 2 | 1 | 1 | 1 | 1 | 2 | >8 | II | Neg | Neg | Blood | No Match |
| MRSA-92 | 1 | 4 | 8 | 1 | 1 | 0.5 | 0.5 | 0.5 | 1 | 2 | >8 | II | Neg | Neg | Blood | USA-100 |
| MRSA-93 | 1 | 2 | 8 | 1 | 1 | 0.5 | 0.5 | 0.5 | 0.5 | 2 | >8 | IV | Neg | Neg | Blood | USA-800 |
| MRSA-94 | 1 | 4 | 4 | 1 | 1 | 0.5 | 0.5 | 0.5 | 1 | 2 | >8 | IV a | Pos | Pos | Blood | USA-300 |
| MRSA-95 | 1 | 4 | 16 | 1 | 1 | 1 | 1 | 1 | 1 | 2 | >8 | II | Neg | Neg | Blood | USA-100 |
| MRSA-96 | 1 | 4 | 8 | 1 | 1 | 0.5 | 0.5 | 0.5 | 0.5 | 2 | >8 | IV | Neg | Neg | Blood | USA-100 |
| MRSA-97 | 1 | 4 | 16 | 1 | 2 | 1 | 1 | 1 | 1 | 2 | >8 | II | Neg | Neg | Blood | USA-100 |
| MRSA-98 | 1 | 4 | 2 | 1 | 1 | 0.5 | 0.5 | 0.5 | 0.5 | 2 | >8 | IV a | Pos | Pos | Blood | USA-300 |
| MRSA-99 | 1 | 4 | 8 | 1 | 2 | 0.5 | 0.5 | 1 | 1 | 2 | >8 | II | Neg | Neg | Blood | USA-100 |
| MRSA-100 | 1 | 4 | 4 | 1 | 1 | 0.5 | 0.5 | 0.5 | 1 | 2 | >8 | IV a | Pos | Pos | Blood | USA-300 |
| MRSA-101 | 1 | 2 | 2 | 1 | 1 | 0.5 | 0.5 | 0.5 | 0.5 | 2 | >8 | IV a | Pos | Pos | Blood | USA-300 |
| MRSA-102 | 1 | 2 | 16 | 1 | 1 | 0.5 | 1 | 1 | 1 | 2 | >8 | IV a | Pos | Pos | Blood | USA-300 |
| MRSA-103 | 1 | 4 | 4 | 1 | 1 | 0.5 | 0.5 | 0.5 | 0.5 | 2 | >8 | IV | Neg | Neg | Blood | No Match |
| MRSA-104 | 1 | 4 | 4 | 1 | 1 | 0.5 | 0.5 | 0.5 | 0.5 | 2 | >8 | IV a | Pos | Pos | Blood | USA-300 |
| MRSA-105 | 1 | 2 | 2 | 1 | 1 | 0.5 | 0.5 | 0.5 | 0.5 | 2 | >8 | IV a | Pos | Pos | Blood | USA-300 |
| MRSA-106 | 1 | 4 | 4 | 1 | 1 | 0.5 | 0.5 | 0.5 | 1 | 2 | >8 | IV a | Pos | Pos | Blood | USA-300 |
| MRSA-107 | 1 | 4 | 4 | 1 | 1 | 0.5 | 0.5 | 1 | 1 | 2 | >8 | II | Neg | Neg | Blood | USA-100 |
| MRSA-108 | 1 | 4 | 1 | 2 | 2 | 4 | 4 | 0.25 | 0.5 | 1 | >8 | IV a | Pos | Pos | Blood | USA-300 |
| MRSA-109 | 1 | 2 | 4 | 0.5 | 0.5 | 0.5 | 0.5 | 1 | 1 | 2 | >8 | II | Neg | Pos | Blood | No Match |
| MRSA-110 | 1 | 4 | 4 | 1 | 1 | 0.5 | 0.5 | 0.5 | 0.5 | 2 | >8 | IV a | Pos | Pos | Blood | USA-300 |
| MRSA-111 | 1 | 4 | 4 | 1 | 1 | 0.5 | 0.5 | 0.5 | 0.5 | 2 | >8 | II | Neg | Neg | Blood | USA-100 |
| MRSA-112 | 1 | 4 | 4 | 2 | 2 | 4 | 4 | 0.5 | 0.5 | 2 | >8 | IV a | Pos | Pos | Blood | USA-300 |
| MRSA-113 | 1 | 2 | 16 | 1 | 1 | 0.5 | 0.5 | 0.5 | 0.5 | 2 | >8 | IV | Neg | Neg | Blood | USA-100 |
| MRSA-114 | 1 | 2 | 4 | 1 | 1 | 0.5 | 0.5 | 0.5 | 0.5 | 1 | >8 | IV a | Pos | Pos | Blood | USA-300 |
| MRSA-115 | 1 | 2 | 8 | 1 | 1 | 0.5 | 0.5 | 1 | 1 | 2 | >8 | II | Neg | Neg | Blood | USA-100 |
| MRSA-116 | 1 | 4 | 4 | 1 | 1 | 0.5 | 0.5 | 0.5 | 0.5 | 2 | >8 | IV a | Pos | Pos | Blood | USA-300 |
| MRSA-117 | 1 | 2 | 4 | 1 | 1 | 0.5 | 0.5 | 1 | 1 | 2 | >8 | II | Neg | Pos | Blood | No Match |
| MRSA-118 | 1 | 2 | 8 | 1 | 1 | 0.5 | 0.5 | 0.5 | 1 | 2 | >8 | II | Neg | Neg | Blood | USA-100 |
| MRSA-119 | 1 | 2 | 8 | 1 | 2 | 1 | 1 | 1 | 1 | 2 | >8 | II | Neg | Neg | Blood | USA-100 |
| MRSA-120 | 1 | 4 | 4 | 1 | 1 | 0.5 | 0.5 | 0.5 | 0.5 | 2 | >8 | IV a | Pos | POS | Blood | USA-300 |
| MRSA-121 | 1 | 2 | 8 | 1 | 1 | 0.5 | 0.5 | 1 | 1 | 2 | >8 | II | Neg | Neg | Blood | USA-100 |
| MRSA-122 | 1 | 2 | 4 | 1 | 1 | 0.5 | 0.5 | 0.5 | 0.5 | 2 | >8 | IV a | Pos | POS | Blood | USA-300 |
| MRSA-123 | 1 | 4 | 8 | 1 | 1 | 0.5 | 0.5 | 0.25 | 0.25 | 4 | >8 | IV | Neg | Neg | Blood | USA-800 |
| MRSA-124 | 1 | 4 | 8 | 1 | 1 | 0.5 | 0.5 | 0.5 | 0.5 | 4 | >8 | IV | Neg | Neg | Blood | USA-100 |
| MRSA-125 | 1 | 2 | 4 | 1 | 1 | 0.5 | 0.5 | 0.5 | 0.5 | 2 | >8 | IV a | Pos | Pos | Blood | USA-300 |
| MRSA-126 | 1 | 4 | 4 | 1 | 1 | 0.5 | 0.5 | 0.5 | 0.5 | 2 | >8 | IV a | Pos | Pos | Blood | USA-300 |
| MRSA-127 | 1 | 8 | 2 | 1 | 1 | 0.5 | 0.5 | 0.5 | 0.5 | 2 | >8 | IV a | Pos | Pos | Blood | USA-300 |
| MRSA-128 | 1 | 2 | 2 | 1 | 1 | 0.5 | 0.5 | 0.5 | 0.5 | 2 | >8 | IV a | Pos | Pos | Blood | USA-300 |
| MRSA-129 | 1 | 2 | 4 | 1 | 1 | 0.25 | 0.25 | 0.5 | 0.5 | 2 | >8 | IV | Neg | Neg | Blood | USA-100 |
| MRSA-130 | 1 | 2 | 8 | 1 | 1 | 0.5 | 0.5 | 0.5 | 0.5 | 2 | >8 | IV | Neg | Neg | Blood | USA-800 |
| MRSA-131 | 1 | 4 | 4 | 1 | 1 | 0.5 | 0.5 | 1 | 1 | 2 | >8 | II | Neg | Neg | Blood | No Match |
| MRSA-132 | 1 | 2 | 4 | 1 | 1 | 0.5 | 0.5 | 0.5 | 1 | 2 | >8 | II | Neg | Neg | Blood | No Match |
| MRSA-133 | 1 | 2 | 4 | 1 | 1 | 0.5 | 0.5 | 0.5 | 0.5 | 2 | >8 | IV a | Pos | Pos | Blood | USA-300 |
| MRSA-134 | 1 | 2 | 8 | 1 | 1 | 0.5 | 0.5 | 1 | 1 | 2 | >8 | II | Neg | Neg | Blood | USA-100 |
| MRSA-135 | 1 | 2 | 8 | 1 | 1 | 0.5 | 0.5 | 0.5 | 0.5 | 2 | >8 | IV | Neg | Neg | Blood | USA-800 |
| MRSA-136 | 1 | 4 | 8 | 1 | 1 | 1 | 1 | 2 | 2 | 2 | >8 | II | Neg | Neg | Blood | USA-100 |
| MRSA-137 | 1 | 2 | 4 | 4 | 4 | 1 | 2 | 0.5 | 1 | 2 | 8 | IV a | Pos | Pos | Blood | USA-300 |
| MRSA-138 | 1 | 1 | 4 | 1 | 1 | 0.25 | 0.5 | 1 | 1 | 2 | >8 | II | Neg | Pos | Blood | USA-100 |
| MRSA-139 | 1 | 4 | 4 | 1 | 1 | 0.5 | 0.5 | 0.5 | 0.5 | 2 | >8 | IV a | Pos | Pos | Blood | USA-300 |
| MRSA-140 | 1 | 2 | 4 | 1 | 1 | 0.5 | 0.5 | 0.5 | 0.5 | 2 | >8 | IV a | Pos | Pos | Blood | USA-300 |
| MRSA-141 | 1 | 2 | 4 | 1 | 1 | 0.5 | 0.5 | 1 | 1 | 4 | >8 | II | Neg | Neg | Blood | USA-100 |
| MRSA-142 | 1 | 2 | 4 | 2 | 2 | 0.5 | 0.5 | 0.5 | 0.5 | 2 | >8 | IV a | Pos | Pos | Blood | USA-300 |
| MRSA-143 | 1 | 2 | 8 | 1 | 1 | 0.5 | 0.5 | 0.5 | 0.5 | 2 | >8 | IV a | Pos | Pos | Blood | USA-300 |
| MRSA-144 | 1 | 2 | 2 | 1 | 1 | 1 | 1 | 0.5 | 0.5 | 2 | >8 | IV a | Pos | Pos | Blood | USA-300 |
| MRSA-145 | 1 | 8 | 8 | 1 | 1 | 0.5 | 0.5 | 0.5 | 0.5 | 2 | >8 | IV | Neg | Neg | Blood | USA-800 |
| MRSA-146 | 1 | 2 | 8 | 1 | 1 | 0.5 | 0.5 | 1 | 1 | 2 | >8 | II | Neg | Neg | Blood | No Match |
| MRSA-147 | 1 | 4 | 8 | 1 | 1 | 0.5 | 0.5 | 0.5 | 0.5 | 2 | >8 | IV a | Pos | Pos | Blood | USA-300 |
| MRSA-148 | 1 | 2 | 4 | 1 | 1 | 0.5 | 0.5 | 0.5 | 0.5 | 2 | >8 | IV a | Neg | Pos | Blood | USA-300 |
| MRSA-149 | 1 | 2 | 4 | 1 | 1 | 0.5 | 0.5 | 1 | 1 | 2 | 0 | II | Neg | Neg | Blood | No Match |
| MRSA-150 | 1 | 2 | 2 | 1 | 1 | 0.5 | 1 | 0.5 | 0.5 | 2 | >8 | IV a | Pos | Pos | Blood | USA-300 |
| MRSA-151 | 1 | 2 | 2 | 1 | 1 | 0.5 | 0.5 | 0.5 | 0.5 | 2 | >8 | IV a | Pos | Pos | Blood | USA-300 |
| MRSA-152 | 1 | 2 | 8 | 1 | 1 | 0.5 | 0.5 | 0.5 | 0.5 | 2 | >8 | IV a | Pos | Pos | Blood | USA-300 |
| MRSA-153 | 1 | 2 | 2 | 1 | 1 | 0.5 | 0.5 | 0.5 | 0.5 | 2 | >8 | IV a | Pos | Pos | Blood | USA-300 |
| MRSA-154 | 1 | 2 | 4 | 1 | 1 | 0.5 | 0.5 | 0.5 | 1 | 2 | >8 | IV a | Pos | Pos | Blood | USA-300 |
| MRSA-155 | 1 | 2 | 4 | 1 | 1 | 0.5 | 1 | 0.5 | 1 | 2 | >8 | II | Neg | Neg | Blood | USA-100 |
| MRSA-156 | 1 | 2 | 8 | 1 | 1 | 0.5 | 0.5 | 0.5 | 0.5 | 2 | >8 | IV | Neg | Neg | Blood | USA-800 |
| MRSA-157 | 1 | 4 | 4 | 1 | 1 | 1 | 1 | 0.5 | 0.5 | 2 | >8 | IV a | Neg | Neg | Blood | USA-300 |
| MRSA-158 | 1 | 2 | 4 | 1 | 1 | 0.5 | 0.5 | 0.5 | 0.5 | 2 | >8 | IV a | Pos | Pos | Blood | USA-300 |
| MRSA-159 | 1 | 2 | 4 | 0.5 | 0.5 | 0.5 | 0.5 | 0.5 | 0.5 | 2 | >8 | IV a | Pos | Neg | Blood | USA-300 |
| MRSA-160 | 1 | 4 | 8 | 1 | 1 | 0.5 | 0.5 | 1 | 1 | 2 | >8 | II | Neg | Neg | Blood | USA-100 |
| MRSA-161 | 1 | 4 | 8 | 1 | 1 | 0.5 | 0.5 | 0.5 | 1 | 2 | >8 | IV a | Pos | Pos | Blood | USA-300 |
| MRSA-162 | 1 | 2 | 4 | 1 | 1 | 0.5 | 0.5 | 0.5 | 0.5 | 2 | >8 | IV a | Pos | pos | Blood | USA-300 |
| MRSA-163 | 1 | 2 | 4 | 1 | 1 | 0.5 | 0.5 | 0.5 | 0.5 | 2 | >8 | IV a | Pos | Pos | Blood | USA-300 |
| MRSA-164 | 1 | 2 | 8 | 1 | 1 | 0.5 | 0.5 | 0.5 | 0.5 | 2 | >8 | IV | Neg | Neg | Blood | USA-100 |
| MRSA-165 | 1 | 2 | 2 | 1 | 1 | 0.5 | 0.5 | 0.5 | 1 | 2 | >8 | IV a | Pos | Pos | Blood | USA-300 |
| MRSA-166 | 1 | 2 | 4 | 1 | 1 | 0.5 | 0.5 | 0.5 | 0.5 | 2 | >8 | IV a | Pos | Pos | Blood | USA-300 |
| MRSA-167 | 1 | 2 | 4 | 1 | 1 | 0.5 | 0.5 | 1 | 1 | 2 | >8 | II | Neg | Pos | Blood | USA-100 |
| MRSA-168 | 1 | 2 | 2 | 1 | 2 | 1 | 1 | 0.5 | 1 | 2 | >8 | IV a | Pos | Pos | Blood | USA-300 |
| MRSA-169 | 1 | 4 | 8 | 1 | 1 | 0.5 | 0.5 | 0.5 | 1 | 2 | >8 | IV | Neg | Pos | Blood | No Match |
| MRSA-170 | 1 | 2 | 4 | 1 | 1 | 0.5 | 0.5 | 0.5 | 0.5 | 2 | >8 | IV a | Pos | Pos | Blood | USA-300 |
| MRSA-171 | 1 | 2 | 4 | 1 | 1 | 0.5 | 0.5 | 0.5 | 0.5 | 2 | >8 | IV a | Pos | Pos | Blood | USA-300 |
| MRSA-172 | 1 | 8 | 4 | 1 | 1 | 2 | 4 | 0.5 | 0.5 | 2 | >8 | IV a | Pos | Pos | Blood | USA-300 |
| MRSA-173 | 1 | 2 | 4 | 1 | 1 | 0.5 | 0.5 | 0.5 | 1 | 2 | >8 | IV a | Pos | Pos | Blood | USA-300 |
| MRSA-174 | 1 | 2 | 4 | 1 | 2 | 0.5 | 0.5 | 0.5 | 1 | 2 | >8 | IV a | Pos | Pos | Blood | USA-300 |
| MRSA-175 | 1 | 2 | 16 | 2 | 2 | 1 | 1 | 0.5 | 0.5 | 2 | >8 | II | Neg | Neg | Blood | USA-100 |
| MRSA-176 | 1 | 2 | 1 | 1 | 2 | 2 | 2 | 1 | 1 | 1 | >8 | II | Neg | Neg | Blood | USA-100 |
| MRSA-177 | 1 | 2 | 4 | 0.5 | 0.5 | 0.5 | 0.5 | 0.5 | 1 | 2 | >8 | IV a | Pos | Pos | Blood | USA-300 |
| MRSA-178 | 1 | 2 | 8 | 1 | 1 | 0.5 | 0.5 | 0.5 | 1 | 2 | >8 | IV a | Pos | Pos | Blood | USA-300 |
| MRSA-179 | 1 | 2 | 4 | 1 | 1 | 0.5 | 0.5 | 0.5 | 0.5 | 2 | >8 | IV a | Pos | Pos | Blood | USA-300 |
| MRSA-180 | 1 | 2 | 8 | 1 | 2 | 0.5 | 0.5 | 0.5 | 0.5 | 2 | >8 | IV | Neg | Pos | Blood | USA-800 |
| MRSA-181 | 1 | 2 | 8 | 1 | 1 | 0.5 | 0.5 | 0.5 | 0.5 | 2 | >8 | IV a | Neg | Pos | Blood | USA-300 |
| MRSA-182 | 1 | 2 | 2 | 1 | 1 | 0.5 | 0.5 | 0.5 | 0.5 | 2 | >8 | IV | Pos | Weak Pos | Blood | USA-300 |
| MRSA-183 | 1 | 4 | 4 | 1 | 1 | 0.5 | 0.5 | 0.25 | 0.5 | 2 | > 8 | IV | Neg | Pos | Blood | USA-100 |
| MRSA-184 | 1 | 4 | 8 | 1 | 1 | 0.5 | 0.5 | 0.5 | 0.5 | 2 | >8 | IV a | Neg | Pos | Blood | USA-300 |
| MRSA-185 | 1 | 2 | 4 | 1 | 1 | 0.5 | 0.5 | 0.5 | 0.5 | 2 | >8 | IV a | Pos | Pos | Blood | USA-300 |
| MRSA-186 | 1 | 4 | 4 | 1 | 1 | 0.5 | 0.5 | 0.5 | 1 | 2 | >8 | IV a | Pos | Pos | Blood | USA-300 |
| MRSA-187 | 1 | 2 | 4 | 1 | 2 | 0.5 | 0.5 | 0.5 | 0.5 | 2 | >8 | IV a | Pos | Pos | Blood | USA-300 |
| MRSA-188 | 1 | 2 | 4 | 1 | 1 | 0.5 | 0.5 | 0.5 | 0.5 | 2 | >8 | IV a | Pos | Pos | Blood | USA-300 |

MIC, Minimum Inhibitory Concentration in mg/L; MBC, Minimum Bactericidal Concentration in mg/L; PFGE, Pulse Field Gel Electrophoresis; PVL, Panton-Valentine Leukocidin; SCC, Staphylococcal Cassette Chromosome; ROM, Rhodomyrtone; FOS, Fosfomycin; VAN, Vancomycin; DAP, Daptomycin; CPT, Ceftaroline; LZD, Linezolid.

Supplemental Table 2: Molecular Characteristics, Source and In Vitro Activity of Rhodomyrtone, Fosfomycin, Vancomycin, Daptomycin, Ceftaroline and Linezolid against VISA study isolates (n=31).

| ID # | ROM | | FOS | | CPT | | VAN | | DAP | | LZD | | SCC | PVL | arc(A) | Source | PFGE Group |
| --- | --- | --- | --- | --- | --- | --- | --- | --- | --- | --- | --- | --- | --- | --- | --- | --- | --- |
|  | MIC | MBC | MIC | MBC | MIC | MBC | MIC | MBC | MIC | MBC | MIC | MBC |  |  |  |  |  |
| NRS-1 | 1 | 2 | >512 | >512 | 1 | 1 | 4 | 4 | 1 | 2 | 2 | 8 | II | Neg | Neg | Mu50 | Unmatched |
| NRS-3 | 1 | 2 | 2 | 4 | 1 | 1 | 8 | 8 | 2 | 2 | 1 | 8 | II | Neg | Neg | Peritoneal Fluid | USA-100 |
| NRS-4 | 1 | 1 | 16 | 16 | 0.5 | 0.5 | 8 | 8 | 0.5 | 1 | 1 | 8 | II | Neg | Neg | Blood | USA-100 |
| NRS-12 | 1 | 2 | 8 | 32 | 0.25 | 0.5 | 4 | 8 | 4 | 4 | 2 | 16 | MSSA | Neg | Neg | Eye | Unmatched |
| NRS-14 | 1 | 2 | 32 | 32 | 0.5 | 0.5 | 4 | 4 | 2 | 4 | 2 | 16 | MSSA | Neg | Neg | Eye | Unmatched |
| NRS-17 | 1 | 2 | 4 | 8 | 1 | 1 | 8 | 8 | 2 | 2 | 2 | 16 | II | Neg | Neg | Blood | USA-100 |
| NRS-18 | 1 | 2 | 4 | 8 | 0.5 | 0.5 | 4 | 8 | 2 | 2 | 2 | 4 | II | Neg | Neg | Wound | USA-100 |
| NRS-19 | 1 | 2 | 1 | 2 | 0.5 | 1 | 4 | 4 | 2 | 2 | 1 | 4 | II | Neg | Neg | Blood | Unmatched |
| NRS-21 | 1 | 1 | 4 | 8 | 0.5 | 0.5 | 4 | 4 | 1 | 1 | 1 | 4 | IV d | Neg | Neg | Blood | USA-500 |
| NRS-22 | 1 | 2 | 1 | 2 | 0.5 | 0.5 | 4 | 4 | 2 | 2 | 2 | 4 | II | Neg | Neg | Blood | USA-600 |
| NRS-23 | 1 | 2 | 8 | 16 | 1 | 1 | 4 | 4 | 2 | 2 | 2 | 16 | II | Neg | Neg | Bone/Joint | USA-100 |
| NRS-24 | 1 | 2 | 8 | 16 | 0.5 | 1 | 4 | 4 | 2 | 2 | 2 | 4 | II | Neg | Neg | Wound | Unmatched |
| NRS-26 | 1 | 4 | 32 | 64 | 0.5 | 0.5 | 8 | 8 | 4 | 8 | 1 | 16 | II | Neg | Neg | Blood | USA-500 |
| NRS-27 | 1 | 8 | 1 | 1 | 0.5 | 1 | 4 | 4 | 2 | 2 | 2 | 4 | II | Neg | Neg | CSF | USA-600 |
| NRS-39 | 1 | 2 | 512 | >512 | 1 | 2 | 8 | 8 | 2 | 2 | 1 | 8 | I | Neg | Neg | Urine | Unmatched |
| NRS-49 | 1 | 2 | >512 | >512 | 1 | 2 | 8 | 8 | 1 | 1 | 2 | 16 | II | Neg | Neg | Unknown | Unmatched |
| NRS-51 | 1 | 4 | 16 | 64 | 1 | 1 | 4 | 4 | 1 | 1 | 4 | 32 | II | Neg | Neg | Bile | Unmatched |
| NRS-52 | 1 | 2 | 16 | 64 | 0.25 | 0.25 | 8 | 8 | 2 | 4 | 2 | 32 | MSSA | Neg | Neg | Bile | Unmatched |
| NRS-54 | 1 | 1 | >512 | >512 | 1 | 1 | 4 | 4 | 1 | 2 | 2 | 32 | III | Neg | Neg | Wound | Unmatched |
| NRS-56 | 1 | 2 | >512 | >512 | 1 | 1 | 4 | 4 | 2 | 2 | 1 | 32 | III | Neg | Neg | Wound | Unmatched |
| NRS-63 | 1 | 2 | 16 | 16 | 0.5 | 0.5 | 4 | 8 | 2 | 2 | 1 | 16 | MSSA | Neg | Neg | Unknown | Unmatched |
| NRS-65 | 1 | 1 | 2 | 4 | 1 | 2 | 4 | 4 | 2 | 2 | 1 | 4 | III | Neg | Neg | Blood | Unmatched |
| NRS-73 | 1 | 2 | 4 | 16 | 0.5 | 0.5 | 4 | 4 | 2 | 2 | 1 | 16 | IV d | Neg | Neg | Wound | USA-500 |
| NRS-74 | 1 | 4 | 32 | 64 | 0.25 | 0.5 | 8 | 8 | 4 | 4 | 2 | 4 | II | Neg | Neg | Blood | Unmatched |
| NRS-76 | 1 | 4 | 2 | 4 | 0.25 | 0.5 | 8 | 8 | 2 | 2 | 2 | 16 | II | Neg | Neg | Blood | USA-100 |
| NRS-118 | 1 | 2 | 2 | 4 | 1 | 2 | 8 | 8 | 2 | 2 | 1 | 4 | I | Neg | Neg | Respiratory | Unmatched |
| NRS-126 | 1 | 1 | 4 | 16 | 1 | 1 | 4 | 4 | 2 | 2 | 2 | 16 | II | Neg | Neg | Blood | USA-100 |
| NRS-272 | 0.5 | 1 | 1 | 16 | 1 | 1 | 4 | 4 | 1 | 2 | 1 | 16 | I | Neg | Neg | Sputum | Unmatched |
| NRS-402 | 1 | 2 | 32 | 128 | 1 | 2 | 8 | 8 | 4 | 4 | 2 | 16 | II | Neg | Neg | Blood | USA-100 |
| NRS-403 | 1 | 2 | 4 | 8 | 0.5 | 1 | 8 | 8 | 2 | 2 | 2 | 4 | II | Neg | Neg | Blood | USA-100 |
| NRS-404 | 0.5 | 1 | 16 | 32 | 0.5 | 1 | 4 | 4 | 2 | 2 | 2 | 8 | II | Neg | Neg | Blood | USA-100 |
| J-31 | 1 | 1 | 2 | 4 | 1 | 1 | 4 | 4 | 8 | 16 | 1 | 4 | III | Neg | Neg | Blood | Unmatched |
| J-32 | 1 | 4 | 8 | 8 | 1 | 1 | 4 | 4 | 2 | 2 | 2 | 8 | II | Neg | Pos | Blood | USA-100 |
| J-33 | 1 | 2 | 8 | 8 | 1 | 1 | 4 | 4 | 0.5 | 1 | 2 | 4 | II | Neg | Neg | Blood | USA-100 |
| MRSA-137 | 1 | 2 | 4 | 8 | 0.5 | 1 | 4 | 4 | 1 | 2 | 2 | 8 | IV a | Pos | Pos | Blood | USA-300 |

MIC, Minimum Inhibitory Concentration in mg/L; MBC, Minimum Bactericidal Concentration in mg/L; PFGE, Pulse Field Gel Electrophoresis; PVL, Panton-Valentine Leukocidin; SCC, Staphylococcal Cassette Chromosome; ROM, Rhodomyrtone; FOS, Fosfomycin; VAN, Vancomycin; DAP, Daptomycin; CPT, Ceftaroline; LZD, Linezolid.

Supplemental Table 3: Molecular Characteristics, Source and In Vitro Activity of Rhodomyrtone, Fosfomycin, Vancomycin, Daptomycin, Ceftaroline and Linezolid against VRSA study isolates (n=15).

| ID # | ROM | | FOS | | CPT | | VAN | | DAP | | LZD | | SCC | PVL | arc(A) | Source | PFGE Group |
| --- | --- | --- | --- | --- | --- | --- | --- | --- | --- | --- | --- | --- | --- | --- | --- | --- | --- |
|  | MIC | MBC | MIC | MBC | MIC | MBC | MIC | MBC | MIC | MBC | MIC | MBC |  |  |  |  |  |
| VRS-1 | 1 | 2 | 2 | 8 | 1 | 1 | >64 | >64 | 0.5 | 0.5 | 2 | 8 | II | Neg | Neg | Catheter Exit | USA-100 |
| VRS-2 | 1 | 2 | 4 | 8 | 0.5 | 1 | 32 | 64 | 0.5 | 0.5 | 2 | 4 | II | Neg | Neg | Plantar Ulcer | USA-100 |
| VRS-3a | 1 | 2 | 2 | 2 | 0.5 | 1 | 32 | 64 | 0.5 | 0.5 | 2 | 8 | IV | Neg | Neg | Urine | USA-800 |
| VRS-3b | 1 | 2 | 2 | 4 | 0.5 | 1 | >64 | >64 | 0.5 | 0.5 | 2 | 8 | IV | Neg | Neg | Nephro tube | USA 800 |
| VRS-4 | 1 | 4 | 4 | 8 | 0.5 | 1 | >64 | >64 | 1 | 1 | 2 | 8 | II | Neg | Neg | Wound | USA-100 |
| VRS-5 | 1 | 4 | 8 | 16 | 0.5 | 0.5 | >64 | >64 | 0.5 | 0.5 | 4 | 32 | II | Neg | Neg | Surgical Site | USA-100 |
| VRS-6 | 1 | 1 | 2 | 4 | 0.25 | 0.25 | >64 | >64 | 0.5 | 1 | 2 | 4 | II | Neg | Neg | Wound | Unmatched |
| VRS-7 | 1 | 1 | 2 | 4 | 0.5 | 1 | >64 | >64 | 0.25 | 0.25 | 1 | 4 | II | Neg | Neg | Wound | Unmatched |
| VRS-8 | 1 | 2 | 2 | 8 | 1 | 1 | >64 | >64 | 0.25 | 0.5 | 2 | 4 | II | Neg | Neg | Wound | USA 100 |
| VRS-9 | 1 | 2 | 8 | 32 | 0.5 | 1 | > 64 | >64 | 0.5 | 0.5 | 2 | 16 | II | Neg | Neg | Wound | USA-100 |
| VRS-10 | 1 | 2 | 4 | 8 | 1 | 1 | >64 | >64 | 1 | 1 | 2 | 32 | II | Neg | Neg | Wound | USA-100 |
| VRS-11a | 0.5 | 1 | 4 | 8 | 0.5 | 0.5 | >64 | >64 | 1 | 1 | 2 | 8 | II | Neg | Neg | Wound Drainage | USA-100 |
| VRS-11b | 0.5 | 1 | 4 | 8 | 0.5 | 0.5 | >64 | >64 | 0.5 | 1 | 2 | 4 | II | Neg | Neg | Wound Drainage | USA-100 |
| VRS-12 | 1 | 4 | 16 | 32 | 0.5 | 0.5 | >64 | >64 | 0.5 | 0.5 | 2 | 32 | II | Neg | Neg | Unknown | Unmatched |
| VRS-13 | 1 | 4 | 8 | 16 | 0.5 | 0.5 | >64 | >64 | 0.25 | 0.25 | 2 | 16 | IV | Neg | Neg | Wound | Unmatched |

MIC, Minimum Inhibitory Concentration in mg/L; MBC, Minimum Bactericidal Concentration in mg/L; PFGE, Pulse Field Gel Electrophoresis; PVL, Panton-Valentine Leukocidin; SCC, Staphylococcal Cassette Chromosome; ROM, Rhodomyrtone; FOS, Fosfomycin; VAN, Vancomycin; DAP, Daptomycin; CPT, Ceftaroline; LZD, Linezolid.

Supplemental Table 4: Molecular Characteristics, Sources, and In Vitro Activity of Rhodomyrtone, Fosfomycin, Vancomycin, Daptomycin, Ceftaroline and Linezolid against LRSA study isolates (n=6).

| ID # | ROM | | FOS | | CPT | | VAN | | DAP | | LZD | | SCC | PVL | arc(A) | Source | PFGE Group |
| --- | --- | --- | --- | --- | --- | --- | --- | --- | --- | --- | --- | --- | --- | --- | --- | --- | --- |
|  | MIC | MBC | MIC | MBC | MIC | MBC | MIC | MBC | MIC | MBC | MIC | MBC |  |  |  |  |  |
| NRS-120 | 0.5 | 2 | 8 | 16 | 1 | 1 | 2 | 2 | 1 | 1 | 64 | >64 | IV d | Neg | Neg | Unknown | USA-500 |
| NRS-121 | 0.5 | 1 | 32 | 32 | 1 | 1 | 2 | 2 | 1 | 1 | 64 | >64 | IV d | Neg | Neg | Unknown | USA-500 |
| NRS-127 | 1 | 2 | 8 | 16 | 0.5 | 1 | 2 | 2 | 0.5 | 0.5 | 16 | 32 | II | Neg | Neg | Sputum | USA-100 |
| NRS-271 | 1 | 2 | 8 | 16 | 1 | 1 | 1 | 1 | 0.5 | 0.5 | 32 | > 64 | IV | Neg | Neg | Wound Drain | Unmatched |
| LRSA-10 | 1 | 4 | 8 | 8 | 0.5 | 1 | 1 | 1 | 0.5 | 0.5 | 16 | >64 | II | Neg | Neg | Blood | Unmatched |
| LRSA-11 | 1 | 2 | 8 | 8 | 1 | 1 | 1 | 1 | 0.5 | 0.5 | 16 | 64 | II | Neg | Neg | Blood | USA-100 |

MIC, Minimum Inhibitory Concentration in mg/L; MBC, Minimum Bactericidal Concentration in mg/L; PFGE, Pulse Field Gel Electrophoresis; PVL, Panton-Valentine Leukocidin; SCC, Staphylococcal Cassette Chromosome; ROM, Rhodomyrtone; FOS, Fosfomycin; VAN, Vancomycin; DAP, Daptomycin; CPT, Ceftaroline; LZD, Linezolid.

Supplemental Table 5: Molecular Characteristics, Source and In Vitro Activity of Rhodomyrtone, Fosfomycin, Vancomycin, Daptomycin, Ceftaroline and Linezolid against DRSA study isolates (n=14).

| ID # | ROM | | FOS | | CPT | | VAN | | DAP | | LZD | | SCC | PVL | arc(A) | Source | PFGE Group |
| --- | --- | --- | --- | --- | --- | --- | --- | --- | --- | --- | --- | --- | --- | --- | --- | --- | --- |
|  | MIC | MBC | MIC | MBC | MIC | MBC | MIC | MBC | MIC | MBC | MIC | MBC |  |  |  |  |  |
| DNS-1 | 1 | 2 | 16 | 16 | 0.5 | 1 | 2 | 2 | 4 | 4 | 2 | 32 | II | Neg | Neg | Blood | USA-100 |
| DNS-2 | 1 | 2 | 8 | 8 | 1 | 1 | 2 | 2 | 8 | 8 | 1 | 16 | II | Neg | Neg | Blood | Unmatched |
| DNS-3 | 0.5 | 1 | 4 | 8 | 1 | 1 | 2 | 2 | 4 | 4 | 2 | 16 | II | Neg | Neg | Blood | USA-100 |
| DNS-4 | 1 | 2 | 8 | 16 | 1 | 1 | 2 | 2 | 2 | 4 | 2 | 32 | III | Neg | Neg | Blood | Unmatched |
| DNS-5 | 0.5 | 2 | 2 | 4 | 0.5 | 0.5 | 2 | 2 | 2 | 2 | 1 | 16 | IV a | Pos | Pos | Blood | USA-300 |
| DNS-6 | 1 | 2 | 2 | 4 | 0.5 | 1 | 2 | 2 | 4 | 4 | 2 | 32 | II | Neg | Neg | Blood | USA-100 |
| DNS-7 | 1 | 1 | 8 | 16 | 0.5 | 1 | 2 | 2 | 2 | 2 | 2 | 32 | IV a | Pos | Pos | Blood | USA-300 |
| DNS-8 | 1 | 8 | 8 | 8 | 0.5 | 0.5 | 2 | 2 | 2 | 4 | 2 | 32 | IV a | Pos | Pos | Blood | USA-300 |
| DNS-9 | 0.5 | 2 | 4 | 8 | 0.5 | 0.5 | 2 | 2 | 2 | 2 | 2 | 32 | II | Neg | Neg | Blood | USA-100 |
| MRSA-63 | 1 | 2 | 8 | 16 | 0.5 | 1 | 2 | 2 | 2 | 2 | 2 | > 8 | IV | Neg | NA | Blood | USA-700 |
| MRSA-108 | 1 | 4 | 1 | 4 | 0.25 | 0.5 | 2 | 2 | 4 | 4 | 1 | > 8 | IV a | Pos | NA | Blood | USA-300 |
| MRSA-112 | 1 | 4 | 4 | 8 | 0.5 | 0.5 | 2 | 2 | 4 | 4 | 2 | > 8 | IV a | Pos | Na | Blood | USA-300 |
| MRSA-172 | 1 | 8 | 4 | 4 | 0.5 | 0.5 | 1 | 1 | 2 | 4 | 2 | > 8 | IV a | Pos | NA | Blood | USA-300 |
| MRSA-176 | 1 | 2 | 2 | 8 | 1 | 1 | 1 | 2 | 2 | 2 | 1 | > 8 | II | Neg | NA | Blood | USA-100 |

MIC, Minimum Inhibitory Concentration in mg/L; MBC, Minimum Bactericidal Concentration in mg/L; PFGE, Pulse Field Gel Electrophoresis; PVL, Panton-Valentine Leukocidin; SCC, Staphylococcal Cassette Chromosome; ROM, Rhodomyrtone; FOS, Fosfomycin; VAN, Vancomycin; DAP, Daptomycin; CPT, Ceftaroline; LZD, Linezolid; NA, Not Available.

Supplemental Table 6: In Vitro Activity of Rhodomyrtone against MRSA.

|  | Run 1 | | Run 2 | | Run 3 | | Run 4 | |  | |
| --- | --- | --- | --- | --- | --- | --- | --- | --- | --- | --- |
| ID # | MIC | MBC | MIC | MBC | MIC | MBC | MIC | MBC | Final MIC | Final MBC |
| MRSA-79 | 1 | 8 | 1 | 8 |  |  |  |  | 1 | 8 |
| MRSA-80 | 1 | 2 | 1 | 2 |  |  |  |  | 1 | 2 |
| MRSA-81 | 1 | 2 | 1 | 2 |  |  |  |  | 1 | 2 |
| MRSA-82 | 1 | 2 | 1 | 1 | 1 | 4 |  |  | 1 | 2 |
| MRSA-83 | 1 | 2 | 1 | 2 |  |  |  |  | 1 | 2 |
| MRSA-84 | 1 | 2 | 1 | 2 |  |  |  |  | 1 | 2 |
| MRSA-85 | 1 | 4 | 1 | 4 |  |  |  |  | 1 | 4 |
| MRSA-86 | 1 | >8 | 1 | 2 | 1 | 2 |  |  | 1 | 2 |
| MRSA-87 | 1 | NA | 1 | 2 | 1 | 2 |  |  | 1 | 2 |
| MRSA-88 | 1 | NA | 1 | 2 | 1 | 2 |  |  | 1 | 2 |
| MRSA-89 | 1 | 2 | 2 | 4 | 1 | 2 |  |  | 1 | 2 |
| MRSA-90 | 1 | 4 | 1 | 2 | 1 | 2 |  |  | 1 | 2 |
| MRSA-91 | 1 | 4 | 1 | 4 |  |  |  |  | 1 | 4 |
| MRSA-92 | 1 | 4 | 1 | 4 |  |  |  |  | 1 | 4 |
| MRSA-93 | 1 | >8 | 2 | 2 | 1 | 2 | 1 | 2 | 1 | 2 |
| MRSA-94 | 1 | 2/ >8 | 2 | 2 | 1 | 4 | 1 | 4 | 1 | 4 |
| MRSA-95 | 1 | 2 | 1 | 4 | 1 | 4 |  |  | 1 | 4 |
| MRSA-96 | 1 | 8 | 1 | 4 | 1 | 2 |  |  | 1 | 4 |
| MRSA-97 | 1 | 8 | 1 | 4 | 1 | 2 |  |  | 1 | 4 |
| MRSA-98 | 1 | 8 | 1 | 4 | 1 | 2 |  |  | 1 | 4 |
| MRSA-99 | 1 | 8 | 1 | 4 | 1 | 2 |  |  | 1 | 4 |
| MRSA-100 | 1 | 4 | 1 | 4 |  |  |  |  | 1 | 4 |
| MRSA-101 | 1 | 2 | 1 | 2 |  |  |  |  | 1 | 2 |
| MRSA-102 | 1 | 2 | 0.5 | 2 | 1 | 2 |  |  | 1 | 2 |
| MRSA-103 | 1 | 4 | 1 | 4 |  |  |  |  | 1 | 4 |
| MRSA-104 | 1 | 4 | 1 | 4 |  |  |  |  | 1 | 4 |
| MRSA-105 | 1 | 2 | 1 | 2 |  |  |  |  | 1 | 2 |
| MRSA-106 | 0.5 | 2 | 1 | 4 | 1 | 4 |  |  | 1 | 4 |
| MRSA-107 | 1 | 4 | 1 | 4 |  |  |  |  | 1 | 4 |
| MRSA-108 | 1 | 4 | 1 | 4 |  |  |  |  | 1 | 4 |
| MRSA-109 | 1 | 2 | 1 | 1 | 1 | 2 |  |  | 1 | 2 |
| MRSA-110 | 1 | 4 | 1 | 4 |  |  |  |  | 1 | 4 |
| MRSA-111 | 1 | 2 | 1 | 8 | 1 | 4 |  |  | 1 | 4 |
| MRSA-112 | 1 | 4 | 1 | 2 | 1 | 4 |  |  | 1 | 4 |
| MRSA-113 | 1 | 4 | 1 | 2 | 1 | 2 |  |  | 1 | 2 |
| MRSA-114 | 1 | 2 | 2 | 4 | 1 | 2 |  |  | 1 | 2 |
| MRSA-115 | 1 | 2 | 1 | 2 |  |  |  |  | 1 | 2 |
| MRSA-116 | 1 | 4 | 1 | 2 | 1 | 4 |  |  | 1 | 4 |
| MRSA-117 | 1 | 2 | 1 | 2 |  |  |  |  | 1 | 2 |
| MRSA-118 | 1 | 2 | 1 | 8 | 1 | 2 |  |  | 1 | 2 |
| MRSA-119 | 1 | 2 | 2 | 2 | 1 | 2 |  |  | 1 | 2 |
| MRSA-120 | 1 | 4 | 1 | >8.0 | 2 | 2 | 1 | 4 | 1 | 4 |
| MRSA-121 | 1 | 2 | 1 | 2 |  |  |  |  | 1 | 2 |
| MRSA-122 | 1 | >8.0 | 1 | 2 | 1 | 8 | 1 | 2 | 1 | 2 |
| MRSA-123 | 1 | 4 | 1 | >8.0 | 1 | 4 |  |  | 1 | 4 |
| MRSA-124 | 1 | 4 | 1 | 2 | 1 | 4 |  |  | 1 | 4 |
| MRSA-125 | 1 | 2 | 1 | 2 |  |  |  |  | 1 | 2 |
| MRSA-126 | 1 | 2 | 1 | 8 | 1 | 4 |  |  | 1 | 4 |
| MRSA-127 | 1 | 8 | 1 | 8 |  |  |  |  | 1 | 8 |
| MRSA-128 | 1 | 2 | 1 | 4 | 1 | 2 |  |  | 1 | 2 |
| MRSA-129 | 1 | 2 | 1 | 1 | 1 | 2 |  |  | 1 | 2 |
| MRSA-130 | 1 | 2 | 1 | 2 |  |  |  |  | 1 | 2 |
| MRSA-131 | 1 | 4 | 1 | 4 |  |  |  |  | 1 | 4 |
| MRSA-132 | 1 | 2 | 1 | 2 |  |  |  |  | 1 | 2 |
| MRSA-133 | 1 | 2 | 1 | 2 |  |  |  |  | 1 | 2 |
| MRSA-134 | 1 | 8 | 1 | 2 | 1 | 2 |  |  | 1 | 2 |
| MRSA-135 | 1 | 8 | 1 | 2 | 1 | 2 |  |  | 1 | 2 |
| MRSA-136 | 1 | 4 | 1 | 4 |  |  |  |  | 1 | 4 |
| MRSA-137 | 1 | 4 | 1 | 2 | 1 | 2 |  |  | 1 | 2 |
| MRSA-138 | 1 | 1 | 1 | 1 |  |  |  |  | 1 | 1 |
| MRSA-139 | 1 | 2 | 1 | 4 | 1 | 4 |  |  | 1 | 4 |
| MRSA-140 | 1 | 2 | 1 | 2 |  |  |  |  | 1 | 2 |
| MRSA-141 | 1 | 2 | 1 | 2 |  |  |  |  | 1 | 2 |
| MRSA-142 | 1 | 2 | 1 | 2 |  |  |  |  | 1 | 2 |
| MRSA-143 | 1 | 2 | 1 | 2 |  |  |  |  | 1 | 2 |
| MRSA-144 | 1 | 2 | 1 | 8 | 1 | 2 |  |  | 1 | 2 |
| MRSA-145 | 1 | 8 | 1 | 8 |  |  |  |  | 1 | 8 |
| MRSA-146 | 1 | 2 | 1 | 2 |  |  |  |  | 1 | 2 |
| MRSA-147 | 1 | 4 | 1 | 4 |  |  |  |  | 1 | 4 |
| MRSA-148 | 1 | 2 | 1 | 2 |  |  |  |  | 1 | 2 |
| MRSA-149 | 1 | 2 | 1 | 2 |  |  |  |  | 1 | 2 |
| MRSA-150 | 1 | 2 | 1 | 2 |  |  |  |  | 1 | 2 |
| MRSA-151 | 1 | 2 | 1 | 8 | 1 | 2 |  |  | 1 | 2 |
| MRSA-152 | 1 | 2 | 1 | 2 |  |  |  |  | 1 | 2 |
| MRSA-153 | 1 | 2 | 1 | 8 | 1 | 2 |  |  | 1 | 2 |
| MRSA-154 | 1 | 2 | 1 | 2 |  |  |  |  | 1 | 2 |
| MRSA-155 | 1 | 2 | 1 | 2 |  |  |  |  | 1 | 2 |
| MRSA-156 | 1 | 4 | 1 | 2 | 1 | 2 |  |  | 1 | 2 |
| MRSA-157 | 1 | 4 | 1 | 4 |  |  |  |  | 1 | 4 |
| MRSA-158 | 1 | 2/4 | 2 | >8.0 | 1 | 2 | 1 | 2 | 1 | 2 |
| MRSA-159 | 1 | 2 | 1 | 1 | 1 | 2 |  |  | 1 | 2 |
| MRSA-160 | 1 | 4 | 1 | 2 | 1 | 8 |  |  | 1 | 4 |
| MRSA-161 | 1 | 4 | 1 | 4 |  |  |  |  | 1 | 4 |
| MRSA-162 | 1 | 2 | 1 | 2 |  |  |  |  | 1 | 2 |
| MRSA-163 | 1 | 2 | 1 | 2 |  |  |  |  | 1 | 2 |
| MRSA-164 | 1 | 2 | 1 | 4 | 1 | 2 |  |  | 1 | 2 |
| MRSA-165 | 1 | 4 | 1 | 2 | 1 | 2 |  |  | 1 | 2 |
| MRSA-166 | 1 | 8 | 1 | 2 | 1 | 2 |  |  | 1 | 2 |
| MRSA-167 | 1 | 2 | 1 | 2 |  |  |  |  | 1 | 2 |
| MRSA-168 | 1 | 2 | 1 | 2 |  |  |  |  | 1 | 2 |
| MRSA-169 | 1 | 8 | 1 | 4 | 1 | 2 |  |  | 1 | 4 |
| MRSA-170 | 1 | 2 | 1 | 2 |  |  |  |  | 1 | 2 |
| MRSA-171 | 1 | 2 | 1 | 4 | 1 | 2 |  |  | 1 | 2 |
| MRSA-172 | 1 | 8 | 1 | 8 |  |  |  |  | 1 | 8 |
| MRSA-173 | 1 | 4 | 1 | 2 | 1 | 1 |  |  | 1 | 2 |
| MRSA-174 | 1 | 2 | 1 | 8 | 1 | 2 |  |  | 1 | 2 |
| MRSA-175 | 1 | 2 | 1 | 2 |  |  |  |  | 1 | 2 |
| MRSA-176 | 1 | 2 | 1 | 2 | 0.5 | 2 |  |  | 1 | 2 |
| MRSA-177 | 1 | 2 | 1 | 2 | 1 | 4 | 1 | 2 | 1 | 2 |
| MRSA-178 | 1 | 2 | 2 | 4 | 1 | 1 | 1 | 4 | 1 | 2 |
| MRSA-179 | 1 | 2 | 1 | 2 |  |  |  |  | 1 | 2 |
| MRSA-180 | 1 | 2 | 1 | 2 |  |  |  |  | 1 | 2 |
| MRSA-181 | 1 | 2 | 2 | 2 | 1 | 2 | 1 | 2 | 1 | 2 |
| MRSA-182 | 1 | 2 | 1 | 8 | 1 | 1 | 1 | 2 | 1 | 2 |
| MRSA-183 | 1 | 8 | 1 | 4 | 1 | 2 |  |  | 1 | 4 |
| MRSA-184 | 1 | 4 | 1 | 2 | 1 | 8 | 1 | 4 | 1 | 4 |
| MRSA-185 | 2 | 2 | 1 | 2 | 1 | 1 | 1 | 4 | 1 | 2 |
| MRSA-186 | 2 | 8 | 1 | 4 | 1 | 2 | 1 | 8 | 1 | 4 |
| MRSA-187 | 1 | 4 | 1 | 2 | 1 | 2 |  |  | 1 | 2 |
| MRSA-188 | 1 | 2 | 1 | 2 | 1 | 1 |  |  | 1 | 2 |

MIC, Minimum Inhibitory Concentration in mg/L; MBC, Minimum Bactericidal Concentration in mg/L; NA, Not Available.

Supplemental Table 7: In Vitro Activity of Rhodomyrtone against VISA.

|  | Run 1 | | Run 2 | | Run 3 | | Run 4 | |  | |
| --- | --- | --- | --- | --- | --- | --- | --- | --- | --- | --- |
| ID # | MIC | MBC | MIC | MBC | MIC | MBC | MIC | MBC | Final MIC | Final MBC |
| NRS-1 | 1 | 2 | 1 | 2 |  |  |  |  | 1 | 2 |
| NRS-3 | 1 | 2 | 1 | 1 | 1 | 4 |  |  | 1 | 2 |
| NRS-4 | 1 | 2 | 1 | 1 | 1 | 1 |  |  | 1 | 1 |
| NRS-12 | 0.5 | 2 | 1 | 2 | 1 | 2 | 1 | 8 | 1 | 2 |
| NRS-14 | 1 | 2 | 1 | 2 |  |  |  |  | 1 | 2 |
| NRS-17 | 1 | 2 | 1 | 2 |  |  |  |  | 1 | 2 |
| NRS-18 | 1 | 2 | 1 | 2 |  |  |  |  | 1 | 2 |
| NRS-19 | 1 | 1 | 1 | 2 | 1 | 2 |  |  | 1 | 2 |
| NRS-21 | 1 | 8 | 1 | 1 | 1 | 1 |  |  | 1 | 1 |
| NRS-22 | 1 | 4 | 1 | 2 | 1 | 1 |  |  | 1 | 2 |
| NRS-23 | 1 | 1 | 1 | 2 | 1 | 2 | 1 | 2 | 1 | 2 |
| NRS-24 | 1 | 2 | 1 | 8 | 1 | 1 |  |  | 1 | 2 |
| NRS-26 | 1 | 4 | 1 | 4 | 1 | 2 |  |  | 1 | 4 |
| NRS-27 | 1 | 8 | 2 | 2 | 1 | 8 |  |  | 1 | 8 |
| NRS-39 | 1 | 2 | 1 | 2 |  |  |  |  | 1 | 2 |
| NRS-49 | 1 | 4 | 1 | 2 | 1 | 1 |  |  | 1 | 2 |
| NRS-51 | 1 | 2 | 1 | 4 | 1 | 4 |  |  | 1 | 4 |
| NRS-52 | 1 | 8 | 1 | 2 | 1 | 1 |  |  | 1 | 2 |
| NRS-54 | 1 | 1 | 1 | 2 | 1 | 1 |  |  | 1 | 1 |
| NRS-56 | 1 | 2 | 1 | 2 | 1 | 2 | 1 | 2 | 1 | 2 |
| NRS-63 | 1 | 2 | 1 | 4 | 1 | 2 |  |  | 1 | 2 |
| NRS-65 | 1 | 4 | 1 | 1 | 1 | 1 |  |  | 1 | 1 |
| NRS-73 | 1 | 2 | 1 | 1 | 1 | >8 | 1 | 4 | 1 | 2 |
| NRS-74 | 1 | 4 | 1 | 2 | 1 | 4 |  |  | 1 | 4 |
| NRS-76 | 1 | 4 | 1 | 4 |  |  |  |  | 1 | 4 |
| NRS-118 | 1 | 2 | 1 | 2 |  |  |  |  | 1 | 2 |
| NRS-126 | 1 | 2 | 1 | 1 | 1 | 1 |  |  | 1 | 1 |
| NRS-272 | 0.5 | 1 | 0.5 | 1 |  |  |  |  | 0.5 | 1 |
| NRS-402 | 1 | 1 | 1 | 4 | 1 | 2 |  |  | 1 | 2 |
| NRS-403 | 1 | 2 | 1 | 2 |  |  |  |  | 1 | 2 |
| NRS-404 | 0.5 | 1 | 0.5 | 1 |  |  |  |  | 0.5 | 1 |
| J-31 | 1 | 2 | 1 | 1 | 1 | 1 |  |  | 1 | 1 |
| J-32 | 1 | 2 | 1 | 4 | 1 | 4 |  |  | 1 | 4 |
| J-33 | 1 | 4 | 1 | 1 | 1 | 2 |  |  | 1 | 2 |
| MRSA 137 | 1 | 4 | 1 | 2 | 1 | 2 |  |  | 1 | 2 |

MIC, Minimum Inhibitory Concentration in mg/L; MBC, Minimum Bactericidal Concentration in mg/L.

Supplemental Table 8: In Vitro Activity of Rhodomyrtone against DRSA.

|  | Run 1 | | Run 2 | | Run 3 | |  | |
| --- | --- | --- | --- | --- | --- | --- | --- | --- |
| ID # | MIC | MBC | MIC | MBC | MIC | MBC | Final MIC | Final MBC |
| DNS-1 | 1 | 2 | 1 | 4 | 1 | 2 | 1 | 2 |
| DNS-2 | 1 | 2 | 1 | 1 | 1 | 2 | 1 | 2 |
| DNS-3 | 0.5 | 1 | 0.5 | 1 |  |  | 0.5 | 1 |
| DNS-4 | 1 | 2 | 1 | 4 | 1 | 2 | 1 | 2 |
| DNS-5 | 0.5 | 1 | 0.5 | 4 | 0.5 | 2 | 0.5 | 2 |
| DNS-6 | 1 | 2 | 1 | 2 |  |  | 1 | 2 |
| DNS-7 | 1 | 1 | 1 | 1 |  |  | 1 | 1 |
| DNS-8 | 1 | 2 | 1 | 8 | 1 | 8 | 1 | 8 |
| DNS-9 | 0.5 | 2 | 0.5 | 4 | 0.5 | 2 | 0.5 | 2 |
| MRSA-63 | 1 | 2 | 1 | 2 |  |  | 1 | 2 |
| MRSA-108 | 1 | 4 | 1 | 4 |  |  | 1 | 4 |
| MRSA-112 | 1 | 4 | 1 | 2 | 1 | 4 | 1 | 4 |
| MRSA-172 | 1 | 8 | 1 | 8 |  |  | 1 | 8 |
| MRSA-176 | 1 | 2 | 1 | 2 | 0.5 | 2 | 1 | 2 |

MIC, Minimum Inhibitory Concentration in mg/L; MBC, Minimum Bactericidal Concentration in mg/L.

Supplemental Table 9: In Vitro Activity of Rhodomyrtone against LRSA.

|  | Run 1 | | Run 2 | | Run 3 | | Run 4 | |  | |
| --- | --- | --- | --- | --- | --- | --- | --- | --- | --- | --- |
| ID # | MIC | MBC | MIC | MBC | MIC | MBC | MIC | MBC | Final MIC | Final MBC |
| NRS-120 | 1 | 4 | 0.5 | 1 | 0.5 | 2 | 0.5 | 4 | 0.5 | 2 |
| NRS-121 | 1 | 2 | 0.5 | 1 | 0.5 | 1 | 0.5 | 2 | 0.5 | 1 |
| NRS-127 | 1 | 2 | 1 | 8 | 1 | 1 | 1 | 2 | 1 | 2 |
| NRS-271 | 1 | 2 | 1 | 2 | 1 | 2 |  |  | 1 | 2 |
| LRSA-10 | 1 | 4 | 1 | > 8 | 1 | 2 |  |  | 1 | 4 |
| LRSA-11 | 1 | 2 | 1 | 1 | 1 | 4 | 1 | 2 | 1 | 2 |

MIC, Minimum Inhibitory Concentration in mg/L; MBC, Minimum Bactericidal Concentration in mg/L.

Supplemental Table 10: In Vitro Activity of Rhodomyrtone against VRSA.

|  | Run 1 | | Run 2 | | Run 3 | | Run 4 | |  | |
| --- | --- | --- | --- | --- | --- | --- | --- | --- | --- | --- |
| ID # | MIC | MBC | MIC | MBC | MIC | MBC | MIC | MBC | Final MIC | Final MBC |
| VRS-1 | 0.5 | 4 | 1 | 2 | 1 | 4 |  |  | 1 | 2 |
| VRS-2 | 0.5 | 8 | 1 | 2 | 1 | 4 | 1 | 2 | 1 | 2 |
| VRS-3a | 1 | 2 | 1 | 2 |  |  |  |  | 1 | 2 |
| VRS-3b | 1 | 2 | 1 | 2 |  |  |  |  | 1 | 2 |
| VRS-4 | 1 | 8 | 1 | 4 | 1 | 2 |  |  | 1 | 4 |
| VRS-5 | 1 | 4 | 1 | 4 |  |  |  |  | 1 | 4 |
| VRS-6 | 1 | 1 | 1 | 1 |  |  |  |  | 1 | 1 |
| VRS-7 | 1 | 1 | 1 | 1 |  |  |  |  | 1 | 1 |
| VRS-8 | 1 | 2 | 1 | 2 |  |  |  |  | 1 | 2 |
| VRS-9 | 1 | 4 | 1 | 2 | 1 | 2 |  |  | 1 | 2 |
| VRS-10 | 1 | 2 | 1 | 2 |  |  |  |  | 1 | 2 |
| VRS-11a | 1 | 4 | 0.5 | 1 | 0.5 | 1 | 1 | 8.0(1.0) | 0.5 | 1 |
| VRS-11b | 1 | 2 | 0.5 | 1 | 0.5 | 2 | 1 | 4.0(1.0) | 0.5 | 1 |
| VRS-12 | 1 | 4 | 1 | 4 |  |  |  |  | 1 | 4 |
| VRS-13 | 1 | >8.0 | 2 | >8.0 | 1 | 4 | 1 | 4 | 1 | 4 |

MIC, Minimum Inhibitory Concentration in mg/L; MBC, Minimum Bactericidal Concentration in mg/L.

# Supplemental Table 11: Staphylococcus aureus (SA) ATCC 29213 Controls in Vitro Activity of Rhodomyrtone.

| Control | Number | MIC | Date |
| --- | --- | --- | --- |
| SA ATCC | 29213 | 1 | 10/5/2022 |
| SA ATCC | 29213 | 1 | 10/5/2022 |
| SA ATCC | 29213 | 1 | 11/30/2022 |
| SA АТСС | 29213 | 1 | 1/5/2023 |
| SA АТСС | 29213 | 1 | 2/1/2023 |
| SA АТСС | 29213 | 1 | 2/22/2023 |
| SA АТСС | 29213 | 1 | 4/6/2023 |
| SA АТСС | 29213 | 1 | 6/8/2023 |
| SA АТСС | 29213 | 1 | 6/29/2023 |
| SA АТСС | 29213 | 1 | 7/20/2023 |
| SA АТСС | 29213 | 1 | 8/10/2023 |
| SA АТСС | 29213 | 1 | 8/24/2023 |
| SA АТСС | 29213 | 1 | 9/13/2023 |
| SA АТСС | 29213 | 1 | 9/28/2023 |
| SA АТСС | 29213 | 1 | 10/12/2023 |

MIC, Minimum Inhibitory Concentration in mg/L.
